# Association of miRNA biosynthesis genes *DROSHA* and *DGCR8* polymorphisms with cancer susceptibility: a systematic review and meta-analysis

**DOI:** 10.1042/BSR20180072

**Published:** 2018-06-27

**Authors:** Jing Wen, Zhi Lv, Hanxi Ding, Xinxin Fang, Mingjun Sun

**Affiliations:** 1 Department of Gastroenterology, The First Affiliated Hospital of China Medical University, Shenyang 110001, China; 2 Department of Anorectal Surgery, The First Affiliated Hospital of China Medical University, Shenyang 110001, China; 3 Tumor Etiology and Screening Department of Cancer Institute and General Surgery, The First Affiliated Hospital of China Medical University and Key Laboratory of Cancer Etiology and Prevention (China Medical University), Liaoning Provincial Education Department, Shenyang 110001, China

**Keywords:** cancer, DROSHA, DGCR8, miRNA, risk, single nucleotide polymorphisms

## Abstract

Single nucleotide polymorphisms (SNPs) in miRNA biosynthesis genes *DROSHA* and *DGCR8* were indicated to be correlated with cancer risk. We comprehensively reviewed and analyzed the effect of *DROSHA* and *DGCR8* polymorphisms on cancer risk. Eligible articles were selected according to a series of inclusion and exclusion criteria. Consequently, ten case–control studies (from nine citations) with 4265 cancer cases and 4349 controls were involved in a meta-analysis of seven most prevalent SNPs (rs10719 T/C, rs6877842 G/C, rs2291109 A/T, rs642321 C/T, rs3757 G/A, rs417309 G/A, rs1640299 T/G). Our findings demonstrated that the rs417309 SNP in *DGCR8* was significantly associated with an elevated risk of overall cancer in every genetic model. In stratified analysis, correlations of *DROSHA* rs10719 and rs6877842 SNPs were observed in Asian and laryngeal cancer subgroups, respectively. Moreover, associations of the rs417309 SNP could also be found in numerous subgroups including: Asian and Caucasian population subgroups; laryngeal and breast cancer subgroups; population-based (PB) and hospital-based (HB) subgroups. In conclusion, the *DROSHA* rs10719, rs6877842 SNPs, and *DGCR8* rs417309 SNP play pivotal roles in cancerogenesis and may be potential biomarkers for cancer-forewarning.

## Introduction

miRNAs are a type of small non-coding RNAs that play roles at post-transcriptional level by sequence-specific binding to the 3′-UTRs of target mRNAs [1]. During the miRNA maturing processing, primary miRNAs (pri-miRNAs) are first synthesized by RNA II polymerase in nucleus. And then, they are converted into precursor miRNAs (pre-miRNAs) by a Drosha–DGCR8 microprocessor complex which is constituted by *DROSHA*, an RNase III superfamily member and its cofactor *DGCR8* [2]. Next, the pre-miRNAs are exported to cytoplasm and converted into mature miRNAs by *DICER*. miRNA genes are deemed to function as both oncogenes and tumor suppressors and their expressions have been confirmed to be associated with varieties of cancers [3–5]. Hence, imparied miRNA processing caused by the aberrant expression of miRNA biosynthesis genes *DROSHA* or *DGCR8* can noticeably promote the tumorigenesis [6].

As the most prevalent genetic variation, single nucleotide polymorphisms (SNPs) in *DROSHA* and *DGCR8* genes can affect their structure or expression, resulting in incomplete miRNA processing and in turn influence the expression of target genes, thereby acting as risk factor for diseases such as cancer. Thus far, accumulating studies have been concerned with the association between *DROSHA* and *DGCR8* SNPs and the susceptibility to cancer. However, the findings were inconsistent and there was no systematic analysis for *DROSHA* and *DGCR8* SNPs and cancer risk. In the present study, we comprehensively reviewed the eligible studies and analyzed all available data. Our aim is to explore the association of *DROSHA* and *DGCR8* SNPs with cancer risk, supplying clues to researchers for screening novel cancer biomarkers.

## Methods

### Retrieval strategy

A detailed literature retrieval was performed by two independent investigators (J.W. and Z.L.) for publications regarding the association between *DROSHA* and *DGCR8* polymorphisms and cancer risk. Relevant publications were selected from PubMed and Web of Science using a combination of the following keywords: ‘*DROSHA*/drosha ribonuclease III/RNase III/*DGCR8*/Digeorge syndrome critical region gene 8/Pasha’; ‘SNP/polymorphism/variation/variant’; and ‘tumor/cancer/carcinoma/neoplasm’, up to 1 January 2018.

### Inclusion and exclusion criteria

Eligible publications were selected by the following inclusion criteria: (i) a case–control designed study; (ii) regarding the correlation between *DROSHA* or *DGCR8* polymorphisms and cancer risk. Articles meeting the following criteria were excluded: (i) reviews, letters, or editorials; (ii) duplicate records; (iii) unrelated to cancer or *DROSHA* and *DGCR8* polymorphisms; (iv) no available data to extract.

### Data extraction

Data extraction was completed by two independent investigators (J.W. and Z.L.). Basic features obtained from each eligible article were as follows: first author’s name, publication year (unpublished collected study year), country, ethnicity, type of cancer, gene, polymorphisms, sample size of cases and controls, genotype distribution, Hardy–Weinberg equilibrium (HWE) in controls, source of control groups (population-based (PB) or hospital-based (HB)), genotyping method, adjusted factors, and quality score. When the article covered multiple stages, data were extracted individually. When the data in eligible articles were unavailable, we tried our best to contact the corresponding authors for original data.

### Methodology quality assessment

Quality of the selected studies was assessed by two independent reviewers (H.D. and X.F.) according to a study regarding the method for assigning quality scores, which was mentioned in prior meta-analyses [7,8]. Six items were evaluated in the quality assessment scale: (i) the representativeness of the cases; (ii) the source of controls; (iii) the ascertainment of relevant cancers; (iv) the sample size; (v) the quality control of the genotyping methods; (vi) HWE in controls. The quality scores of eligible studies ranged from 0 to 10. Studies with scores less than 5 and HWE disequilibrium were removed from the subsequent analyses.

### Statistical analysis

All statistical analyses in the present study were performed by STATA software, version 11.0 (STATA Corp, College Station, TX, U.S.A.). All statistical tests presented were two-tailed and the *P*-values<0.05 were regarded as statistically significant, unless highlighted otherwise. And the Bonferroni correction was conducted to justify *P*-values [40]. The HWE for the genotype frequencies of *DROSHA* and *DGCR8* polymorphisms in controls was computed by χ^2^ test. The intensity of the correlations between the *DROSHA* and *DGCR8* polymorphisms and the risk of cancer was estimated by odds ratios (ORs) with its corresponding 95% confidence intervals (95% CIs). Between-study heterogeneity was computed by a χ^2^-based Cochran’s *Q*test (significance at *P*<0.10 and *I^2^* > 50%). We summarized the results by using fixed effect models [9] when the interstudy heterogeneity was absent, otherwise random effect models [10]. Begg’s test and Egger’s linear regression analysis were performed to estimate the publication bias statistically [11,12]. *P*<0.10 was regarded as statistically significant in both Egger’s and Begg’s test [8,28]. What is more, sensitivity analysis was shown to inspect whether the pooled results were steady after we excluded the outlying studies.

## Results

### Characteristics of the included studies

**Figure 1:**
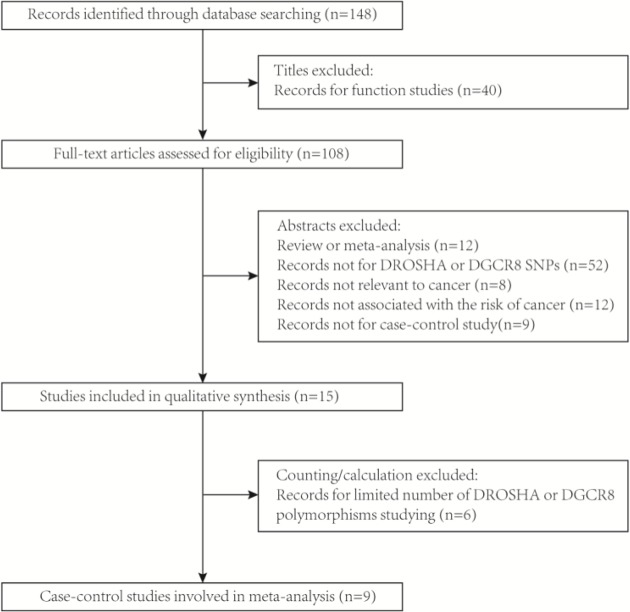
The flow chart of identification for studies included in the meta-analysis

**Figure 2:**
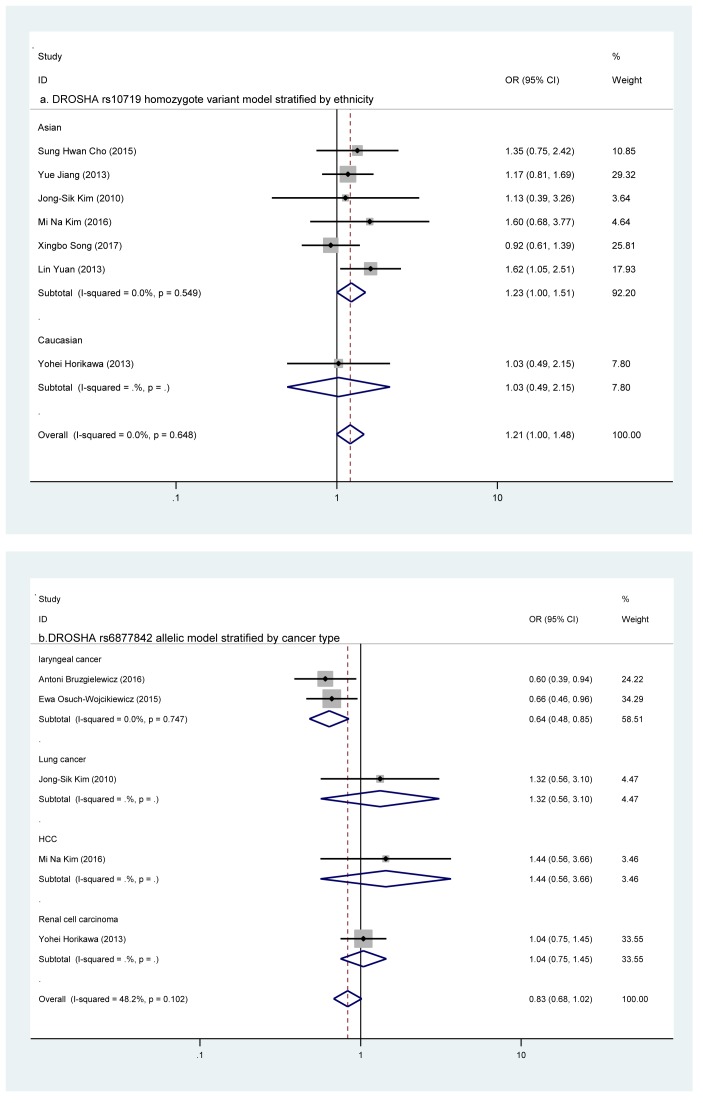

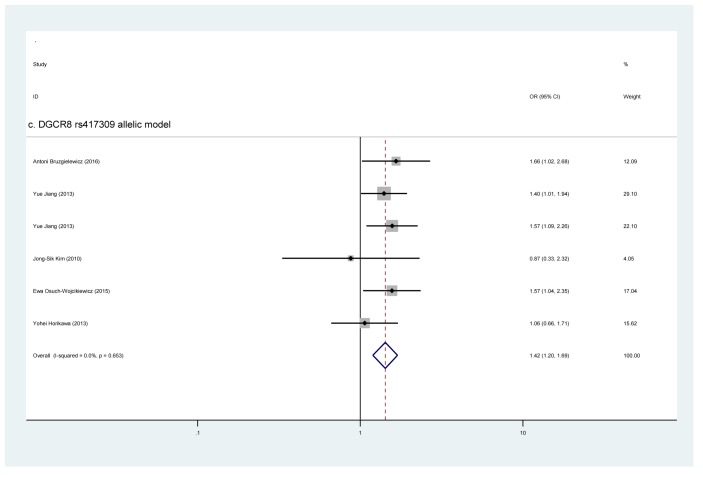
A forest plot of the *DROSHA* and *DGCR8* SNPs associated with cancer risk ((a) DROSHA rs10719 in the ethnicity subgroup analysis; (b) DROSHA rs6877842 in the cancer type subgroups; (c) DGCR8 rs417309 under allelic model: A compared with G).

As presented in Figure 1, a total of 148 publications were collected through database search after eliminating the duplicate studies. We eliminated 83 records after browsing the titles and abstracts (40 were functional studies; 12 were reviews or meta-analysis; 9 were not case–control studies; 52 were unrelated to *DROSHA* or *DGCR8* SNPs; 8 were unrelated to cancer; 12 were not correlated with cancer risks). What was more, six studies were excluded by calculating (one for the unavailable data; five for the limited study number of *DROSHA* or *DGCR8* polymorphisms). Moreover, the removal of two records from the subsequent analyses was due to the inconformity of their genotype distributions to HWE (*P*_HWE_<0.05). Hence, in total, ten case–control studies (from nine citations) containing 4265 cancer cases and 4349 cancer-free controls were involved in our meta-analyses, which were accorded with our inclusion criteria and the evaluation of methodology quality. The characteristics of these involved articles were presented in Table 1 and the frequency distributions of *DROSHA* and *DGCR8* polymorphisms genotype were shown in Table 2. In summary obtained from ten eligible case–control studies, seven SNPs of *DROSHA* or *DGCR8* genes were investigated in the eventual analysis. According to the SNPs selection criteria mentioned in eligible studies [2,14], we found that none of these seven SNPs were in strong linkage disequilibrium (*r^2^* ≥ 0.8). In *DROSHA*, the analyzed SNPs were rs10719 T/C, rs6877842 G/C, rs2291109 A/T, rs642321 C/T; in *DGCR8*, the analyzed SNPs are rs3757 G/A, rs417309 G/A, rs1640299 T/G (Figure 2).

**Table 1:** The main features of enrolled studies

Ref. no.	Year	Country	Ethnicity	Sample size		Source of controls	Genotyping method	Adjusted factors	Quality score	Citation
				Case	Control					
1	2010	Korean	Asian	93	93	HB	MS	NM	5.5	[15]
2	2013	China	Asian	878	900	PB	Taqman	Age and residential area	7.5	[2]
3	2013	China	Asian	914	967	PB	Taqman	Age and residential area	7.5	[2]
4	2013	China	Asian	685	730	HB	Taqman	Age, sex, and smoking status	7	[16]
5	2013	America	Caucasian	277	278	PB	SNPlex technology	Age, sex, ethnicity, and county of residence	7.5	[14]
6	2015	Korean	Asian	408	400	HB	PCR-RFLP	Age, gender, hypertension, diabetes mellitus	7	[24]
7	2015	Polish	Caucasian	135	170	HB	Taqman	NM	7.5	[25]
8	2016	Polish	Caucasian	100	100	NM	Taqman	NM	6	[13]
9	2016	Korean	Asian	147	209	HB	PCR-RFLP	Age, gender, hypertension, diabetes mellitus, drinking status, and smoking	7	[26]
10	2017	China	Asian	628	502	HB	HRM	Age, sex, region, smoking status, and drinking status	7	[27]

Abbreviations: HRM, high-resolution melting; MS, sequenome MS-based genotyping assay; PCR-RFLP, polymerase chain reaction-restriction fragment length polymorphism; NM, not mentioned.

**Table 2:** Genotype frequency distributions of DROSHA and DGCR8 SNPs in included studies

Ref. No.	Year	Cancer type	Gene	SNPs^1^	Sample size		Case			Control			*P*_HWE_	MAF in controls (Global MAF^4^)	Included in meta-analysis
Case	Control	Homozygote wild	Heterozygote	Homozygote variant	Homozygote wild	Heterozygote	Homozygote variant								
1	2010	Lung cancer	DROSHA	rs6877842	93	93	81	11	1	84	8	1	0.136	0.054 (0.138)	Yes
				(G > C)											
		Lung cancer	DROSHA	rs10719	97	97	59	29	9	52	38	7	0.987	0.268 (0.483)	Yes
				(T > C)											
		Lung cancer	DGCR8	rs3757	94	90	60	27	7	60	24	6	0.114	0.200 (0.182)	Yes
				(G > A)											
		Lung cancer	DGCR8	rs417309	98	97	90	8	0	88	9	0	0.632	0.046 (0.043)	Yes
				(G > A)											
		Lung cancer	DGCR8	rs1640299	98	97	58	33	7	52	40	5	0.444	0.258 (0.381)	Yes
				(T > G)											
2	2013	Breast cancer	DROSHA	rs10719	847	878	433	346	68	463	353	62	0.635	0.272 (0.483)	Yes
				(T > C)											
		Breast cancer	DROSHA	rs17409893	849	885	527	287	35	575	276	34	0.902	0.194 (0.222)	No^3^
				(A > G)											
		Breast cancer	DROSHA	rs2291109	858	886	552	273	33	535	306	45	0.884	0.223 (0.061)	Yes
				(A > T)											
		Breast cancer	DROSHA	rs642321	854	883	212	423	219	231	433	219	0.571	0.493 (0.322)	Yes
				(C > T)											
		Breast cancer	DGCR8	rs1640299	849	891	465	330	54	476	357	58	0.412	0.265 (0.381)	Yes
				(T > G)											
		Breast cancer	DGCR8	rs417309	860	893	771	89	0	826	67	0	0.244	0.038 (0.043)	Yes
				(G > A)											
		Breast cancer	DGCR8	rs720012	867	891	225	425	217	240	451	200	0.668	0.478 (0.221)	No^3^
				(G > A)											
		Breast cancer	DGCR8	rs720014	836	880	542	264	30	555	287	38	0.907	0.206 (0.183)	No^3^
				(T > C)											
3	2013	Breast cancer	DROSHA	rs2291109	899	957	563	296	40	625	298	34	0.835	0.191 (0.061)	Yes
				(A > T)											
		Breast cancer	DGCR8	rs417309	901	960	830	68	3	910	49	1	0.687	0.027 (0.043)	Yes
				(G > A)											
4	2013	Bladder cancer	DROSHA	rs2291109	685	730	421	228	36	419	280	31	0.062	0.234 (0.061)	Yes
				(A > T)											
		Bladder cancer	DROSHA	rs10719	684	727	352	278	54	413	275	39	0.437	0.243 (0.483)	Yes
				(T > C)											
		Bladder cancer	DROSHA	rs642321	685	730	197	326	162	176	371	183	0.655	0.505 (0.322)	Yes
				(C > T)											
5	2013	Renal cell carcinoma	DROSHA	rs10719	252	246	161	75	16	155	76	15	0.177	0.215 (0.483)	Yes
				(T > C)											
		Renal cell carcinoma	DROSHA	rs6877842	275	278	200	65	10	204	65	9	0.185	0.149 (0.138)	Yes
				(G > C)											
		Renal cell carcinoma	DGCR8	rs3757	276	278	163	102	11	162	102	14	0.688	0.234 (0.182)	Yes
				(G > A)											
		Renal cell carcinoma	DGCR8	rs417309	277	278	243	30	4	243	34	1	0.87	0.065 (0.043)	Yes
				(G > A)											
		Renal cell carcinoma	DGCR8	rs1640299	277	278	61	151	65	75	136	67	0.729	0.486 (0.381)	Yes
				(T > G)											
6	2015	Colorectal cancer	DROSHA	rs10719	408	400	224	154	30	211	168	21	0.09	0.263 (0.483)	Yes
				(T > C)											
7	2015	Laryngeal cancer	DROSHA	rs6877842	128	170	73	49	6	76	79	15	0.384	0.321 (0.138)	Yes
				(G > C)											
		Laryngeal cancer	DGCR8	rs417309	112	170	67	32	13	116	46	8	0.227	0.182 (0.043)	Yes
				(G > A)											
		Laryngeal cancer	DGCR8	rs1640299	113	170	60	47	6	61	93	16	**0.021**	0.368 (0.381)	No^2^
				(T > G)											
		Laryngeal cancer	DGCR8	rs3757	122	170	29	89	4	36	119	15	**<0.001**	0.438 (0.182)	No^2^
				(G > A)											
8	2016	Laryngeal cancer	DROSHA	rs6877842	100	100	60	35	5	44	47	9	0.476	0.325 (0.138)	Yes
				(G > C)											
		Laryngeal cancer	DGCR8	rs417309	100	100	60	28	12	69	27	4	0.516	0.175 (0.043)	Yes
				(G > A)											
		Laryngeal cancer	DGCR8	rs1640299	100	100	52	42	6	36	55	9	0.062	0.365 (0.381)	Yes
				(T > G)											
9	2016	Hepatocellular carcinoma	DROSHA	rs10719	147	209	81	53	13	110	88	11	0.215	0.263 (0.483)	Yes
				(T > C)											
		Hepatocellular carcinoma	DROSHA	rs6877842	147	209	138	9	0	200	9	0	0.75	0.022 (0.138)	Yes
				(G > C)											
10	2017	Gastirc cancer	DROSHA	rs10719	628	502	314	257	57	248	205	49	0.487	0.302 (0.483)	Yes
				(T > C)											

The results were in bold if *P*<0.05. Abbreviations: MAF, minor allele frequency; *P*_HWE_, the *P*-value for HWE in control groups. ^1^, The ancestral alleles were referenced in the NCBI database. ^2^, Excluded due to the SNP not being in accordance with HWE. ^3^, Excluded due to the limited number for this locus; ^4^, The global MAFs were referenced in the NCBI database.

### Quantitative data synthesis of seven SNPs in *DROSHA* and *DGCR8* genes

#### Four SNPs in DROSHA

First, all eligible articles were summarized to evaluate the correlation strength of each *DROSHA* SNP with the risk of overall cancer. However, these four SNPs (rs10719 T/C, rs6877842 G/C, rs642321 C/T, and rs2291109 A/T) did not manifest any significant associations with cancer risk in any genetic models (Table 3). Due to the existence of interstudy heterogeneity, stratified analyses were performed.

**Table 3:** Meta-analysis of the association between DROSHA and DGCR8 polymorphisms and cancer risk

		Heterozygote compared with homozygote wild			Homozygote variant compared with homozygote wild			Dominant model			Recessive model			Allelic model		
SNPs	*n*	*P (P_corr_)*	OR (95% CI)	*I^2^* (%)	*P (P_corr_)*	OR (95% CI)	*I^2^* (%)	*P (P_corr_)*	OR (95% CI)	*I^2^* (%)	*P (P_corr_)*	OR (95% CI)	*I^2^* (%)	*P (P_corr_)*	OR (95% CI)	*I^2^* (%)
**DROSHA rs10719 (T > C)**	7	0.934	1.004 (0.904–1.117)	0.2	0.055	1.214 (0.996–1.480)	0.0	0.502	1.035 (0.936–1.145)	10.5	0.053	1.210 (0.998–1.467)	0.0	0.179	1.056 (0.975–1.145)	0
Ethnicity																
Asian	6	0.874	1.009 (0.904–1.126)	15.6	**0.048** (0.336)	1.230 (1.001–1.511)	0.0	0.449	1.041 (0.938–1.157)	10.5	**0.04** (0.336)	1.223 (1.002–1.494)	0.0	0.154	1.062 (0.978–1.154)	0
Caucasian	1	0.796	0.950 (0.645–1.400)	NA	0.944	1.027 (0.491–2.148)	NA	0.838	0.963 (0.668–1.387)	NA	0.907	1.044 (0.504–2.161)	NA	0.904	0.981 (0.725–1.329)	NA
Source of controls																
HB	5	0.905	0.992 (0.869–1.132)	30.1	0.071	1.257 (0.981–1.610)	0	0.643	1.030 (0.908–1.169)	27.2	0.061	1.259 (0.989–1.602)	0	0.25	1.060 (0.0960–1.172)	16.7
PB	2	0.767	1.027 (0.861–1.225)	0	0.396	1.142 (0.822–1.588)	0.0	0.616	1.044 (0.883–1.234)	0	0.463	1.128 (0.818–1.555)	0.0	0.398	1.049 (0.918-1.199)	0
**DROSHA rs6877842 (G > C)**	5	0.174	0.841 (0.655–1.079)	39.3	0.101	0.627 (0.358–1.096)	0.0	0.358^1^	0.839 (0.577–1.220)	50.7	0.218	0.706 (0.406–1.228)	0.0	0.073	0.832 (0.680–1.017)	48.2
Ethnicity																
Asian	2	0.292	1.438 (0.732–2.824)	0	0.98	1.037 (0.064–16.860)	NA	0.303	1.414 (0.731–2.735)	0.0	1	1.000 (0.062–16.230)	NA	0.326	1.372 (0.731–2.576)	0
Caucasian	3	0.059	0.772 (0.590–1.010)	47.1	0.094	0.613 (0.346–1.087)	27.5	0.125^1^	0.716 (0.467–1.097)	61.1	0.21	0.697 (0.396–1.225)	0.0	0.123^1^	0.762 (0.540–1.076)	60.1
Source of controls																
HB	3	0.395	0.845 (0.574–1.245)	44.3	0.103	0.460 (0.181–1.169)	0	0.88^1^	0.953 (0.506–1.793)	52.5	0.194	0.545 (0.218–1.361)	0	0.155	0.796 (0.581–1.090)	48.0
PB	1	0.922	1.020 (0.687–1.514)	NA	0.79	1.133 (0.451–2.848)	NA	0.862	1.034 (0.710–1.505)	NA	0.797	1.128 (0.451–2.820)	NA	0.807	1.042 (0.750–1.447)	NA
NM	1	0.043	0.546 (0.304–0.981)	NA	0.129	0.407 (0.128–1.300)	NA	0.024	0.524 (0.299–0.919)	NA	0.274	0.532 (0.172–1.648)	NA	0.026	0.603 (0.387–0.941)	NA
Cancer type																
Laryngeal cancer	2	**0.008** (0.56)	0.604 (0.417–0.875)	0	**0.022** (0.154)	0.413 (0.193–0.881)	0.0	**0.002 (0.014)**	0.573 (0.401–0.819)	0.0	0.081	0.518 (0.247–1.084)	0.0	**0.002 (0.014)**	0.638 (0.481–0.847)	0.0
Lung cancer	1	0.469	1.426 (0.546–3.726)	NA	0.98	1.037 (0.064–16.860)	NA	0.488	1.383 (0.553–3.458)	NA	1	1.000 (0.062–16.230)	NA	0.52	1.323 (0.565–3.096)	NA
Hepatocellular carcinoma	1	0.443	1.449 (0.561–3.744)	NA	NA	NA	NA	0.443	1.449 (0.561–3.744)	NA	NA	NA	NA	0.45	1.435 (0.563–3.660)	NA
Renal cell carcinoma	1	0.922	1.020 (0.687–1.514)	NA	0.79	1.133 (0.451–2.848)	NA	0.862	1.034 (0.710–1.505)	NA	0.797	1.128 (0.451–2.820)	NA	0.807	1.042 (0.750–1.447)	NA
**DROSHA rs642321 (C > T)**	2	0.576	0.918 (0.682–1.237)	0.0	0.672^1^	0.934 (0.683–1.279)	60.5	0.603^1^	0.923 (0.681–1.250)	72.2	0.911	0.991 (0.843–1.165)	0	0.665^1^	0.965 (0.821–1.134)	62.3
**DROSHA rs2291109 (A > T)**	3	0.389^1^	0.922 (0.765–1.110)	58.8	0.92	1.014 (0.771–1.333)	44.7	0.469^1^	0.932 (0.770–1.128)	63.8	0.746	1.046 (0.798–1.371)	37.4	0.606^1^	0.958 (0.815–1.126)	64.6
Cancer type																
Breast cancer	2	0.851^1^	0.977 (0.770–1.240)	64.9	0.899^1^	0.962 (0.530–1.747)	69.2	0.859^1^	0.975 (0.738–1.289)	76.4	0.909^1^	0.971 (0.580–1.624)	59.6	0.871^1^	0.978 (0.752–1.272)	81
Bladder cancer	1	0.062	0.810 (0.650–1.011)	NA	0.57	1.156 (0.702–1.903)	NA	0.12	0.845 (0.683–1.045)	NA	0.373	1.251 (0.765–2.046)	NA	0.332	0.917 (0.768–1.093)	NA
Source of controls																
PB	2	0.851^1^	0.977 (0.770–1.240)	64.9	0.899^1^	0.962 (0.530–1.747)	69.2	0.859^1^	0.975 (0.738–1.289)	76.4	0.909^1^	0.971 (0.580–1.624)	59.6	0.871^1^	0.978 (0.752–1.272)	81
HB	1	0.062	0.810 (0.650–1.011)	NA	0.57	1.156 (0.702–1.903)	NA	0.12	0.845 (0.683–1.045)	NA	0.373	1.251 (0.765–2.046)	NA	0.332	0.917 (0.768–1.093)	NA
**DGCR8 rs3757 (G > A)**	2	0.892	1.022 (0.750–1.391)	0.0	0.741	0.894 (0.460–1.737)	0	0.974	1.005 (0.748–1.350)	0	0.715	0.885 (0.460–1.704)	0	0.913	0.986 (0.772–1.260)	0.0
**DGCR8 rs417309 (G > A)**	6	**0.012** (0.084)	1.282 (1.057–1.555)	0.0	**0.001 (0.007)**	3.169 (1.634–6.146)	0	**0.001 0.007)**	1.365 (1.131–1.647)	0	**0.001 (0.007)**	3.026 (1.574–5.817)	0	**6.90E-05 (4.83E-04)**	1.423 (1.196–1.693)	0.0
Ethnicity																
Asian	3	**0.004 (0.028)**	1.420 (1.115–1.809)	0.0	0.303	3.289 (0.341–31.682)	NA	**0.003 0.021)**	1.435 (1.129–1.825)	0	0.314	3.204 (0.333–30.856)	NA	**0.003 (0.021)**	1.429 (1.131–1.806)	0.0
Caucasian	3	0.699	1.066 (0.772–1.472)	0.0	**0.001 (0.007)**	3.157 (1.579–6.310)	0	0.133	1.260 (0.932–1.704)	0	**0.002 (0.014)**	3.009 (1.520–5.954)	0	**0.009** (0.063)	1.415 (1.092–1.834)	2.7
Cancer type																
laryngeal cancer	2	0.387	1.199 (0.795–1.810)	0.0	**0.003 (0.021)**	3.059 (1.474–6.351)	0	0.05	1.460 (1.000–2.131)	0	**0.004 (0.028)**	2.895 (1.410–5.945)	0	**0.003 (0.021)**	1.604 (1.176–2.188)	0.0
Breast cancer	2	**0.003 (0.021)**	1.465 (1.141–1.881)	0.0	0.303	3.289 (0.341–31.682)	NA	**0.002 (0.014)**	1.481 (1.155–1.898)	0	0.314	3.204 (0.333–30.856)	NA	**0.002 (0.014)**	1.473 (1.157–1.876)	0.0
Lung cancer	1	0.783	0.869 (0.321–2.355)	NA	NA	NA	NA	0.783	0.869 (0.321–2.355)	NA	NA	NA	NA	0.788	0.875 (0.330–2.316)	NA
Renal cell carcinoma	1	0.638	0.882 (0.523–1.487)	NA	0.217	4.000 (0.444–36.046)	NA	0.91	0.971 (0.587–1.609)	NA	0.212	4.059 (0.451–36.544)	NA	0.797	1.064 (0.664–1.705)	NA
Source of controls																
PB	3	**0.012** (0.084)	1.333 (1.065–1.669)	33.7	0.107	3.652 (0.755–17.659)	0	**0.006 (0.042)**	1.364 (1.093–1.704)	12.4	**0.036 (0.252)**	2.659 (1.064–6.643)	NA	0.059	1.434 (0.986–2.085)	14.7
HB	2	0.648	1.117 (0.694–1.799)	0.0	**0.029 (0.203)**	2.813 (1.109–7.135)	NA	0.243	1.302 (0.836–2.030)	0	0.108	3.635 (0.752–17.564)	0	**0.003 (0.021)**	1.377 (1.111–1.707)	0.0
NM	1	0.585	1.193 (0.634–2.243)	NA	0.04	3.450 (1.057–11.265)	NA	0.184	1.484 (0.828–2.658)	NA	0.047	3.273 (1.018–10.523)	NA	0.04	1.656 (1.022–2.684)	NA
**DGCR8 rs1640299 (T > G)**	4	0.508^1^	0.895 (0.645–1.243)	60.2	0.988	0.998 (0.751–1.325)	0	0.494	0.898 (0.659–1.223)	59.1	0.786	0.965 (0.745–1.249)	0	0.48	0.959 (0.852–1.078)	34.6
Ethnicity																
Asian	2	0.403	0.923 (0.766–1.113)	0.0	0.91	0.979 (0.674–1.420)	0	0.434	0.931 (0.778–1.114)	0	0.952	1.011 (0.703–1.455)	0	0.545	0.957 (0.829–1.104)	0.0
Caucasian	2	0.769^1^	0.870 (0.344–2.202)	85.3	0.726^1^	0.853 (0.350–2.076)	57.3	0.713^1^	0.844 (0.342–2.085)	85.6	0.656	0.920 (0.638–1.328)	0	0.576^1^	0.864 (0.517–1.443)	77.9
Source of controls																
PB	2	0.643^1^	1.086 (0.767–1.537)	60.2	0.788	1.043 (0.770–1.412)	0	0.694^1^	1.063 (0.785–1.439)	53.5	0.831	0.971 (0.738–1.276)	0	0.974	0.998 (0.879–1.133)	0.0
HB	1	0.32	0.740 (0.408–1.339)	NA	0.712	1.255 (0.375–4.197)	NA	0.433	0.797 (0.452–1.405)	NA	0.565	1.415 (0.433–4.623)	NA	0.682	0.908 (0.574-–1.438)	NA
NM	1	0.033	0.529 (0.295–0.949)	NA	0.175	0.462 (0.151–1.410)	NA	0.023	0.519 (0.295–0.915)	NA	0.424	0.645 (0.221–1.886)	NA	0.042	0.643 (0.421–0.984)	NA

The results are in bold if *P*<0.05. Abbreviation: *P*_corr_, *P*-values after Bonferroni correction. ^1^, *P* was calculated by random model.

In subgroup analyses, rs10719 and rs6877842 SNPs were analyzed in ‘ethnicity’ subgroup; the rs6877842 and rs2291109 SNPs were analyzed in ‘cancer type’ subgroup; rs10719, rs6877842, and rs2291109 SNPs were analyzed in ’source of controls’ subgroup. For rs10719 T/C SNP, its homozygote variant genotype and recessive models were correlated with an elevated cancer risk in Asian (CC compared with TT: OR = 1.230, 95% CI = 1.001–1.511, *P*=0.048; CC + CT compared with TT: OR = 1.223, 95% CI = 1.002–1.494, *P*=0.048, Table 3). For rs6877842 G/C SNP, its heterozygote model had strong correlation with a reduced risk of laryngeal cancer (CG compared with GG: OR = 0.413, 95% CI = 0.193–0.881, *P*=0.022, Table 3) and its homozygote variant genotype, dominant and allelic models had moderate associations with a descending risk of laryngeal cancer (CC compared with GG: OR = 0.604, 95% CI = 0.417–0.875, *P*=0.008; CC compared with CG + GG: OR = 0.573, 95% CI = 0.401–0.819, *P*=0.002; C compared with G: OR = 0.638, 95% CI = 0.481–0.847, *P*=0.002, Table 3). For rs2291109 polymorphism, however, the correlations with cancer risk were not elucidated in any stratified analyses.

#### Three SNPs in DGCR8

We evaluated the correlation strength of the polymorphisms in *DROSHA* gene with cancer risk, based on the entire population. The rs417309 G/A SNP was demonstrated to be associated with an increased risk of cancer. Strong associations of rs417309 were found in homozygote variant genotype and recessive models (AA compared with GG: OR = 3.169, 95% CI = 1.634–6.146, *P*=0.001; AA + AG compared with GG: OR = 3.026, 95% CI = 1.574–5.817, *P*=0.001). Correlations of rs417309 could also be found in other three models (AG compared with GG: OR = 1.282, 95% CI = 1.057–1.555, *P*=0.012; AA compared with AG + GG: OR = 1.365, 95% CI = 1.131–1.647, *P*=0.001; A compared with G: OR = 1.423, 95% CI = 1.196–1.693, *P*<0.001, Table 3). Associations of the rs3757 G/A and rs1640299 T/G SNPs with cancer risk were not illustrated in primary analyses.

In stratified analyses, rs417309 and rs1640299 SNPs were analyzed in ‘ethnicity’ and ‘source of controls’ subgroups; the rs417309 SNP was also analyzed in ‘cancer type’ subgroup. For rs417309 G/A SNP, its associations were observed in every subgroup (including: Asian population, Caucasian population; laryngeal cancer, breast cancer; PB, HB). Amongst all significant associations in subgroup analyses, only strong associations were reported below. In Caucasian subgroup, strong correlations were indicated in homozygote variant genotype and recessive models (AA compared with GG: OR = 3.169, 95% CI = 1.634–6.146, *P*=0.001; AA + AG compared with GG: OR = 3.026, 95% CI = 1.574–5.817, *P*=0.001). In laryngeal cancer subgroup, strong relationships were observed in homozygote variant genotype and recessive models (AA compared with GG: OR = 3.169, 95% CI = 1.634–6.146, *P*=0.001; AA + AG compared with GG: OR = 3.026, 95% CI = 1.574–5.817, *P*=0.001), When the control groups were PB, the recessive type (AA + AG) showed a strong relationship with an increased risk of cancer, compared with the wild-type GG (OR = 1.604, 95% CI = 1.176–2.188, *P*=0.003). When the controls groups were HB, the homozygote variant model of rs417309 presented a strong correlation with cancer risk (AA compared with GG: OR = 2.813, 95% CI = 1.109–7.135, *P*=0.029, Table 3). For rs1640299 T/G polymorphism, however, no significant relationship was found in any subgroup analyses.

#### Sensitivity analysis

Sensitivity analyses were conducted to calculate the effect of individual study on the merged findings by evaluating the sensitivity before and after eliminating each study from our meta-analysis (Supplementary Table S1). For rs417309 SNP, it was no longer statistically significant after we removed the study conducted by Jiang et al. (Supplementary Table S1) [2].

#### Publication bias

Begg’s and Egger’s tests were performed to evaluate the potential publication bias. The publication bias was revealed in the heterozygote genotype and the dominant models of rs10719 SNP in both Begg’s and Egger’s tests, for *P*<0.1 (Table 4). This might be due to the language bias, the lack of publications with opposing results, and/or the inflated estimates caused by a deficient methodological design in smaller studies [1].

**Table 4:** The results of Begg’s and Egger’s tests for the publication bias

	Begg’s test		Egger’s test	
Comparison type	Z-value	*P*-value	*t-*value	*P*-value
**DROSHA rs10719 (T > C)**				
Heterozygote compared with homozygote wild	−2.250	**0.024**	−3.030	**0.029**
Homozygote variant compared with homozygote wild	0.450	0.652	0.300	0.774
Dominant model	−1.950	**0.051**	−2.340	**0.066**
Recessive model	0.560	0.573	1.100	0.332
Allelic model	−0.750	0.453	−1.330	0.241
**DROSHA rs6877842 (G > C)**				
Heterozygote compared with homozygote wild	−0.490	0.624	0.620	0.581
Homozygote variant compared with homozygote wild	0.000	1.000	0.020	0.988
Dominant model	0.000	1.000	0.500	0.650
Recessive model	0.000	1.000	0.030	0.976
Allelic model	0.000	1.000	0.740	0.511
**DROSHA rs642321 (C > T)**				
Heterozygote compared with homozygote wild	−1.000	0.317	NA	NA
Homozygote variant compared with homozygote wild	−1.000	0.317	NA	NA
Dominant model	−1.000	0.317	NA	NA
Recessive model	−1.000	0.317	NA	NA
Allelic model	−1.000	0.317	NA	NA
**DROSHA rs2291109 (A > T)**				
Heterozygote compared with homozygote wild	−1.570	0.117	−1.270	0.426
Homozygote variant compared with homozygote wild	0.520	0.602	0.560	0.673
Dominant model	−0.520	0.602	−0.830	0.558
Recessive model	0.520	0.602	0.870	0.545
Allelic model	−0.520	0.602	−0.430	0.741
**DGCR8 rs3757 (G > A)**				
Heterozygote compared with homozygote wild	1.000	0.317	NA	NA
Homozygote variant compared with homozygote wild	1.000	0.317	NA	NA
Dominant model	1.000	0.317	NA	NA
Recessive model	1.000	0.317	NA	NA
Allelic model	1.000	0.317	NA	NA
**DGCR8 rs417309 (G > A)**				
Heterozygote compared with homozygote wild	−0.940	0.348	−2.150	0.098
Homozygote variant compared with homozygote wild	0.680	0.497	1.460	0.282
Dominant model	−1.320	0.188	−1.460	0.219
Recessive model	0.680	0.497	1.710	0.230
Allelic model	−0.190	0.851	−1.320	0.258
**DGCR8 rs1640299 (T > G)**				
Heterozygote compared with homozygote wild	−0.680	0.497	−0.540	0.644
Homozygote variant compared with homozygote wild	0.000	1.000	−0.560	0.635
Dominant model	−0.680	0.497	−0.560	0.634
Recessive model	0.000	1.000	−0.070	0.949
Allelic model	−0.680	0.497	−0.880	0.471

The results are in bold if *P*<0.1. Abbreviation: NA, not available.

## Discussion

In the present study, total seven SNPs in *DROSHA* and *DGCR8* genes were comprehensively reviewed and analyzed to estimate their associations with the risk of overall cancer. Of these seven SNPs, four (rs6877842, rs642321, rs2291109, rs3757) were analyzed for the first time. Our findings indicated that rs417309 SNP of *DGCR8* might facilitate the cancerogenesis. Moreover, correlations with cancer risk could also be observed in stratified analyses of *DROSHA* rs10719, rs6877842 SNPs and *DGCR8* rs417309 SNP. No associations were revealed amongst other studied SNPs.

### Polymorphisms in DROSHA

As an RNase III superfamily member, *DROSHA* initiates miRNA processing by converting pri-miRNA into pre-miRNA. Current studies have indicated the role of *DROSHA* on the development of several sorts of cancers such as laryngeal, bladder, lung, and so on [13–16]. And mounting studies have focussed on the correlations of *DROSHA* polymorphisms with cancer risk. Based on our analyses, the significant associations with cancer risk could be observed in rs10719 and rs6877842 SNPs.

Regarding rs10719 T/C polymorphism, we presented significant associations between rs10719 SNP (CC or TC + CC genotypes) and cancer risk in Asian population. Located in the *DROSHA* 3′-UTR region, the T to C substitution of rs10719 disrupted an hsa-*miR-27b* binding site, which was identified by luciferase reported gene assays, leading to an overexpression of *DROSHA* gene at the post-transcriptional level [16]. The overexpression of *DROSHA* caused by rs10719-C allele was elucidated to facilitate the proliferation and inhibit apoptosis of cancer cells [17–19], which was in-line with our meta-analysis findings. The meta-analysis of rs10719 analyzed six case–control studies, five of which, however, were inconsistent with our study. From our viewpoint, this phenomenon might be owing to the limitation of sample size, diversity of cancer type, and/or complexity of environmental factors. Hence, further investigations that concentrate on rs10719 SNP are extremely needed to obtain more credible results.

As for rs6877842 G/C polymorphism, strong/moderate correlations with the laryngeal cancer could be observed in every genetic model except recessive model and the rs687742-C allele manifested a protective effect on laryngeal cancer. Located in the promoter region of DROSHA, the rs6877842 SNP might influence the expression level of *DROSHA* by altering the transcription factor binding sites, which was forecasted by a bioinformatics website ‘https://snpinfo.niehs.nih.gov/’, thus inhibiting the laryngeal cancer development. Our study analyzed five case–control studies on different cancers: laryngeal (two), lung (one), hepatocellular (one), and renal cell carcinoma (one). Interestingly, the association of rs6877842 SNP could only be observed in laryngeal cancer subgroup, rather than in the overall cancer analysis. And the between-study heterogeneity was absent after we conducted the ‘cancer type’ subgroup. Thus, it is reasonable to suggest that the effect of rs6877842 SNP on overall cancer susceptibility could be masked by the existence of heterogeneity deriving from different types of cancer. Further investigations on this SNP are in demand to verify our speculation.

### Polymorphisms in DGCR8

The Drosha–DGCR8 microprocessor complex could mediate the biogenesis from pri-miRNA to pre-miRNA, whereas neither Drosha or recombinant DGCR8 alone is active in this processing, suggesting that both the proteins are indispensable in miRNA maturing processing [20]. *DGCR8* are also referred to as Pasha, stabilizes Drosha by protein–protein, and takes charge of recognizing ssRNA and dsRNA structures [21]. Studies have revealed the up-regulation of *DGCR8* expression in various cancers [22,23]. And accumulating researches have focussed on the associations between the *DGCR8* polymorphisms and cancer risk.

The rs417309 G/A polymorphism was the most extensively investigated one amongst *DGCR8* SNPs, and the rs417309-A allele was strongly associated with an elevated cancer susceptibility. Based on the bioinformatics website prediction ‘https://snpinfo.niehs.nih.gov/’, rs417909 SNP was located at miRNA-binding sites (*miR-106b* and *miR-579*) in 3′-UTR region of DGCR8. The risk allele rs417309-A could elevate *DGCR8* expression level, probably through interrupting miRNA binding [2], thus facilitating the cancer development. The meta-analysis of rs417309 SNP involved six case–control studies. Two of them, however, showed no association with cancer risk. Thus, further investigations concerned with rs417309 SNP remain in strong demand for identifying this potential cancer biomarker.

Limitations in our meta-analysis must be recognized. First, only eligible articles published in English were incorporated in our study, which might result in certain publication bias. Second, studies of *DROSHA* and *DGCR8* polymorphisms on cancer predisposition field remain emerging, which resulted in limited number of the relevant investigations. Third, we did not analyze the association of polymorphisms in other miRNA-machinery genes, which were listed in Table 5 including: *DICER1, XPO5, RAN, TARBP2, AGO2, HIWI, GEMIN3*, and *GEMIN4*. Because their study number was limited or because they have already been analyzed in other meta-analyses.

**Table 5:** Reviews of the other miRNA-machinery gene polymorphisms studied in regard to cancer risk

Gene	SNP	Position	Cancer type	Citation
*XPO5*	rs11077 (A > G)	3′-UTR	EC, BC, CRC, TC, RCC, bladder, and larynx cancer	[2,14,15,25,29–34]
*RAN*	rs14035 (C > T)	3′-UTR	RCC, LC, CRC, EC, GC, OC, and larynx cancer	[14,15,24,25,29,32–35]
	rs3803012 (A > G)	3′-UTR	BC, HNC, CC, and HCC	[2,36–38]
	rs3809142 (C > T)	Upstream	BC	[2]
	rs7301722 (C > A)	Upstream	BC	[2]
	rs7958223 (C > A)	Intron	BC	[31]
	rs10848236 (G > A)	Intron	BC	[31]
DICER1	rs1057035 (T > C)	3′-UTR	BC	[2,31]
	rs3742330 (A > G)	3′UTR	PC, LC, and larynx cancer	[15,25,39]
	rs2282265 (A > G)	Intron	BC	[31]
	rs13078 (T > A)	3′-UTR	LC and larynx	[15,25]
TARBP2	rs784567 (A > G)	5′-UTR	PC and larynx	[25]
	rs2280448 (C > T)	5′-UTR	BC	[31]
AGO1	rs595055 (G > A)	Intron	BC	[31]
	rs11263833 (T > G)	Intron	BC	[31]
	rs636832 (A > G)	Intron	LC	[15]
	rs595961 (G > A)	Intron	LC and RCC	[14,15]
AGO2	rs4961280 (A > C)	Upstream	PC	[39]
	rs77216619 (G/T)	Intron	BC	[2]
	rs78796470 (C > T)	Intron	BC	[2]
	rs2292779 (G > C)	Intron	BC	[31]
	rs3864659 (A > C)	Intron	BC	[31]
	rs7016981 (T > C)	Intron	BC	[31]
	rs7824304 (C > T)	Intron	BC	[31]
	rs11786030 (A > G)	3′-UTR	BC	[31]
HIWI	rs10773771 (T > C)	3′-UTR	BC	[2]
	rs4759659 (G > A)	Intron	BC	[31]
	rs7963072 (G > A)	Intron	BC	[31]
	rs1106042 (G > A)	Exon (K527R)	BC and LC	[15,31]
	rs11060845 (G > T)	Intron	BC	[31]
GEMIN3	rs197414 (C > A)	Exon (S693R)	PC, BC, and LC	[15]
	rs197388 (T > A)	Upstream	LC	[15]
	rs197412 (T > C)	Exon (T636I)	LC, RCC, and OC	[14,15,35]
	rs11584657 (C > T)	Upstream	BC	[2]
	rs17504173 (A > G)	3′-UTR	BC	[2]
	rs197413 (G > A)	Exon (V642V)	BC	[31]
	rs17569368 (A > T)	Intron	BC	[31]
GEMIN4	rs7813 (C > T)	Exon (C1033R)	PC,BC, LC, and RCC	[14,15,31]
	rs3744741 (C > T)	Exon (R684Q)	BC and LC	[2,15,31]
	rs4968104 (T > A)	Exon (V593E)	BC and LC	[2,15,31]
	rs2251689 (G > A)	Upstream	BC	[2]
	rs2740348 (C > G)	Exon (E450Q)	BC and LC	[15]
	rs910924 (C > T)	Upstream	LC	[15]
	rs910925 (G > C)	Exon (G579A)	LC	[15]
	rs1062923 (T > C)	Exon (T739I)	LC	[15]
	rs2740349 (A > G)	Exon (N929D)	BC	[31]
FMR1	rs25704 (T > C)	3′-UTR	BC	[31]
	rs28900 (A > C)	Intron	BC	[31]
	rs971000 (C > T)	Intron	BC	[31]

Abbreviations: BC, breast cancer; CC, cervical cancer; CRC, colorectal cancer; EC, esophageal cancer; GC, gastric cancer; HCC, hepatocellular carcinoma; HNC, head and neck cancer; LC, lung cancer; OC, oral cancer; PC, prostate cancer; RCC, renal cell carcinoma; TC, thyroid cancer.

In summary, we performed a systematic review on the association between *DROSHA* and *DGCR8* polymorphisms and risk of cancer. Meanwhile, all available data were utilized to achieve a meta-analysis for seven prevalent SNPs. Three of them (*DROSHA* rs10719, rs6877842, and *DGCR8* rs417309) were revealed to be associated with risk of cancer in whole population or some particular subgroups. Our study generalized the status quo of the current studies on cancer-related polymorphisms in *DROSHA* and *DGCR8* genes, supplying investigators with novel clues for identifying new biomarkers with cancer-forewarning function.

## Supporting information

**Table S1:** ORs (95%CIs) of sensitivity analysis

## Abbreviations

HB: hospital-based
HWE: Hardy–Weinberg equilibrium
OR: odds ratio
PB: population-based
pre-miRNA: precursor miRNA
pri-miRNA: primary miRNA
SNP: single nucleotide polymorphism
95% CI: 95% confidence interval

## References

[1] Xu Q., Liu J.W. and Yuan Y. (2015) Comprehensive assessment of the association between miRNA polymorphisms and gastric cancer risk. Mutat. Res. Rev. Mutat. Res. 763, 148–160 10.1016/j.mrrev.2014.09.004 25795117

[2] Jiang Y. (2013) Evaluation of genetic variants in microRNA biosynthesis genes and risk of breast cancer in Chinese women. Int. J. Cancer 133, 2216–2224 10.1002/ijc.28237 23629745

[3] He L. (2005) A microRNA polycistron as a potential human oncogene. Nature 435, 828–833 10.1038/nature03552 15944707 PMC4599349

[4] Iorio M.V. (2005) MicroRNA gene expression deregulation in human breast cancer. Cancer Res. 65, 7065–7070 10.1158/0008-5472.CAN-05-1783 16103053

[5] Takamizawa J. (2004) Reduced expression of the let-7 microRNAs in human lung cancers in association with shortened postoperative survival. Cancer Res. 64, 3753–3756 10.1158/0008-5472.CAN-04-0637 15172979

[6] Kumar M.S. (2007) Impaired microRNA processing enhances cellular transformation and tumorigenesis. Nat. Genet. 39, 673–677 10.1038/ng2003 17401365

[7] Li Y., Zhang F. and Yang D. (2017) Comprehensive assessment and meta-analysis of the association between CTNNB1 polymorphisms and cancer risk. Biosci. Rep. 37, 10.1042/BSR20171121 PMC5700267 28963373

[8] Lv Z., Xu Q. and Yuan Y. (2017) A systematic review and meta-analysis of the association between long non-coding RNA polymorphisms and cancer risk. Mutat. Res. 771, 1–14 10.1016/j.mrrev.2016.10.002 28342449

[9] Mantel N. and Haenszel W. (1959) Statistical aspects of the analysis of data from retrospective studies of disease. J. Natl. Cancer Inst. 22, 719–748 13655060

[10] DerSimonian R. and Laird N. (1986) Meta-analysis in clinical trials. Control. Clin. Trials 7, 177–188 10.1016/0197-2456(86)90046-2 3802833

[11] Egger M. (1997) Bias in meta-analysis detected by a simple, graphical test. BMJ 315, 629–634 10.1136/bmj.315.7109.629 9310563 PMC2127453

[12] Harbord R.M., Egger M. and Sterne J.A. (2006) A modified test for small-study effects in meta-analyses of controlled trials with binary endpoints. Stat. Med. 25, 3443–3457 10.1002/sim.2380 16345038

[13] Bruzgielewicz A. (2016) Evaluation of polymorphisms in microRNA biosynthesis genes and risk of laryngeal cancer in the Polish population. Pol. J. Pathol. 67, 283–290 10.5114/pjp.2016.63781 28155978

[14] Horikawa Y. (2008) Single nucleotide polymorphisms of microRNA machinery genes modify the risk of renal cell carcinoma. Clin. Cancer Res. 14, 7956–7962 10.1158/1078-0432.CCR-08-1199 19047128 PMC2650498

[15] Kim J.S. (2010) Association of a common AGO1 variant with lung cancer risk: a two-stage case-control study. Mol. Carcinog. 49, 913–921 10.1002/mc.20672 20721975

[16] Yuan L. (2013) Genetic variation in DROSHA 3′UTR regulated by hsa-*miR-27b* is associated with bladder cancer risk. PLoS One 8, e81524 10.1371/journal.pone.0081524 24312312 PMC3842954

[17] Dedes K.J. (2011) Down-regulation of the miRNA master regulators Drosha and Dicer is associated with specific subgroups of breast cancer. Eur. J. Cancer 47, 138–150 10.1016/j.ejca.2010.08.007 20832293

[18] Han Y. (2013) Inducing cell proliferation inhibition and apoptosis via silencing Dicer, Drosha, and Exportin 5 in urothelial carcinoma of the bladder. J. Surg. Oncol. 107, 201–205 10.1002/jso.23214 22766726

[19] Sugito N. (2006) RNASEN regulates cell proliferation and affects survival in esophageal cancer patients. Clin. Cancer Res. 12, 7322–7328 10.1158/1078-0432.CCR-06-0515 17121874

[20] Han J. (2006) Molecular basis for the recognition of primary microRNAs by the Drosha-DGCR8 complex. Cell 125, 887–901 10.1016/j.cell.2006.03.043 16751099

[21] Han J. (2009) Posttranscriptional crossregulation between Drosha and DGCR8. Cell 136, 75–84 10.1016/j.cell.2008.10.053 19135890 PMC2680683

[22] Kim B. (2014) An essential microRNA maturing microprocessor complex component DGCR8 is up-regulated in colorectal carcinomas. Clin. Exp. Med. 14, 331–336 10.1007/s10238-013-0243-8 23775303 PMC4113675

[23] Sand M. (2012) Expression levels of the microRNA maturing microprocessor complex component DGCR8 and the RNA-induced silencing complex (RISC) components argonaute-1, argonaute-2, PACT, TARBP1, and TARBP2 in epithelial skin cancer. Mol. Carcinog. 51, 916–922 10.1002/mc.20861 22025453

[24] Cho S.H. (2015) 3′-UTR polymorphisms in the MiRNA machinery genes DROSHA, DICER1, RAN, and XPO5 are associated with colorectal cancer risk in a Korean population. PLoS ONE 10, e0131125 10.1371/journal.pone.0131125 26147304 PMC4492935

[25] Osuch-Wojcikiewicz E. (2015) Association of polymorphic variants of miRNA processing genes with larynx cancer risk in a Polish population. Biomed. Res. Int. 2015, 298378 10.1155/2015/298378 26688807 PMC4673325

[26] Kim M.N. (2016) Variation in the Dicer and RAN genes are associated with survival in patients with hepatocellular carcinoma. PLoS ONE 11, e0162279 10.1371/journal.pone.0162279 27611467 PMC5017754

[27] Song X. (2017) Association between SNPs in microRNA machinery genes and gastric cancer susceptibility, invasion, and metastasis in Chinese Han population. Oncotarget 8, 86435–86446 10.18632/oncotarget.21199 29156806 PMC5689696

[28] Zhang B. (2011) Genetic variants associated with breast-cancer risk: comprehensive research synopsis, meta-analysis, and epidemiological evidence. Lancet Oncol. 12, 477–488 10.1016/S1470-2045(11)70076-6 21514219 PMC3114278

[29] Yang H. (2008) Evaluation of genetic variants in microRNA-related genes and risk of bladder cancer. Cancer Res. 68, 2530–2537 10.1158/0008-5472.CAN-07-5991 18381463

[30] Ye Y. (2008) Genetic variations in microRNA-related genes are novel susceptibility loci for esophageal cancer risk. Cancer Prev. Res. (Phila.) 1, 460–469 10.1158/1940-6207.CAPR-08-0135 19138993 PMC2768267

[31] Sung H. (2011) Common genetic polymorphisms of microRNA biogenesis pathway genes and risk of breast cancer: a case-control study in Korea. Breast Cancer Res. Treat. 130, 939–951 10.1007/s10549-011-1656-2 21766210

[32] Xie Y. (2015) Single-nucleotide polymorphisms of microRNA processing machinery genes are associated with risk for gastric cancer. Onco. Targets Ther. 8, 567–571 25784816 10.2147/OTT.S79150 PMC4356688

[33] Zhao Y. (2015) Single-nucleotide polymorphisms of microRNA processing machinery genes and risk of colorectal cancer. Onco. Targets Ther. 8, 421–425 25709475 10.2147/OTT.S78647 PMC4334349

[34] Buas M.F. (2015) MiRNA-related SNPs and risk of esophageal adenocarcinoma and Barrett’s esophagus: post genome-wide association analysis in the BEACON consortium. PLoS ONE 10, e0128617 10.1371/journal.pone.0128617 26039359 PMC4454432

[35] Roy R. (2014) Genetic variations at microRNA and processing genes and risk of oral cancer. Tumour Biol. 35, 3409–3414 10.1007/s13277-013-1450-3 24297336

[36] Ma H. (2012) Genetic variations in key microRNA processing genes and risk of head and neck cancer: a case-control study in Chinese population. PLoS ONE 7, e47544 10.1371/journal.pone.0047544 23071822 PMC3469541

[37] Chen J. (2013) Genetic variants in RAN, DICER and HIWI of microRNA biogenesis genes and risk of cervical carcinoma in a Chinese population. Chin. J. Cancer Res. 25, 565–571 24255581 10.3978/j.issn.1000-9604.2013.10.03 PMC3828428

[38] Liu L. (2013) Potentially functional genetic variants in microRNA processing genes and risk of HBV-related hepatocellular carcinoma. Mol. Carcinog. 52, E148–E154 10.1002/mc.22062 23868705

[39] Nikolic Z. (2017) Genetic variants in RNA-induced silencing complex genes and prostate cancer. World J. Urol. 35, 613–624 10.1007/s00345-016-1917-0 27498138

[40] Lv Z. (2017) Long non-coding RNA polymorphisms in 6p21.1 are associated with atrophic gastritis risk and gastric cancer prognosis. Oncotarget 8, 95303–95315 10.18632/oncotarget.20115 29221129 PMC5707023

