# Supplementary material for: Association of miRNA biosynthesis genes DROSHA and DGCR8 polymorphisms with cancer susceptibility: a systematic review and meta-analysis
Source: Biosci Rep. 2018 Jun 27;38(3):BSR20180072. doi: 10.1042/BSR20180072 (PMC6019356; doi:10.1042/BSR20180072)
Supplement: Supplementary file 1 [file bsr20180072_Supp1.pdf]

Table S1. ORs (95%CIs) of sensitivity analysis

| Excluding literature one by one | Heterozygote vs. Homozygote wild | Homozygote variant vs. Homozygote wild | Dominant model | Recessive model | Allelic model |
|---|---|---|---|---|---|
| | OR (95%CI) | OR (95%CI) | OR (95%CI) | OR (95%CI) | OR (95%CI) |
| DROSHA rs10719(T>C) | | | | | |
| Overall | 1.004(0.904-1.117) | 1.214(0.996-1.480) | 1.035(0.936-1.145) | 1.210(0.998-1.467) | 1.056(0.975-1.145) |
| Sung Hwan Cho | 1.028(0.918-1.152) | 1.198(0.971-1.479) | 1.055(0.946-1.175) | 1.184(0.965-1.453) | 1.065(0.978-1.161) |
| Yue Jiang | 0.987(0.871-1.119) | 1.231(0.974-1.557) | 1.023(0.908-1.152) | 1.236(0.983-1.553) | 1.052(0.957-1.157) |
| Jong-Sik Kim | 1.017(0.913-1.133) | 1.217(0.995-1.489) | 1.046(0.944-1.159) | 1.206(0.991-1.468) | 1.063(0.980-1.153) |
| Mi Na Kim | 1.017(0.912-1.134) | 1.195(0.975-1.465) | 1.044(0.941-1.158) | 1.185(0.972-1.445) | 1.058(0.974-1.149) |
| Xingbo Song | 1.008(0.896-1.133) | 1.317(1.051-1.650) | 1.049(0.938-1.173) | 1.312(1.053-1.635) | 1.079(0.986-1.179) |
| Yohei Horikawa | 1.009(0.904-1.126) | 1.230(1.001-1.511) | 1.041(0.938-1.157) | 1.223(1.002-1.494) | 1.062(0.978-1.154) |
| Lin Yuan | 0.955(0.846-1.078) | 1.125(0.900-1.405) | 0.980(0.874-1.100) | 1.142(0.920-1.417) | 1.011(0.923-1.108) |
| DROSHA rs6877842(G>C) | | | | | |
| Overall | 0.841(0.655-1.079) | 0.627(0.358-1.096) | 0.839(0.577-1.220) | 0.706(0.406-1.228) | 0.832(0.680-1.017) |
| Antoni Bruzgielewicz | 0.927(0.703-1.222) | 0.716(0.377-1.360) | 0.944(0.641-1.389) | 0.775(0.410-1.463) | 0.905(0.721-1.135) |
| Jong-Sik Kim | 0.809(0.624-1.048) | 0.613(0.346-1.087) | 0.784(0.523-1.176) | 0.697(0.396-1.225) | 0.809(0.657-0.996) |
| Mi Na Kim | 0.808(0.624-1.046) | 0.627(0.358-1.096) | 0.782(0.525-1.165) | 0.706(0.406-1.228) | 0.810(0.659-0.996) |
| Ewa Osuch-Wojcikiewicz | 0.928(0.693-1.244) | 0.772(0.389-1.532) | 0.939(0.599-1.472) | 0.841(0.426-1.659) | 0.920(0.722-1.172) |
| Yohei Horikawa | 0.740(0.536-1.021) | 0.439(0.212-0.909) | 0.777(0.487-1.242) | 0.540(0.265-1.100) | 0.726(0.561-0.938) |
| DROSHA rs642321(C>T) | | | | | |

| | | | | | |
|---|---|---|---|---|---|
| Overall | 0.918(0.682-1.237) | 0.934(0.683-1.279) | 0.923(0.681-1.250) | 0.991(0.843-1.165) | 0.965(0.821-1.134) |
| Yue Jiang | 0.785(0.610-1.010) | 0.791(0.590-1.060) | 0.787(0.621-0.997) | 0.926(0.726-1.181) | 0.886(0.764-1.026) |
| Lin Yuan | 1.064(0.846-1.339) | 1.090(0.837-1.419) | 1.073(0.865-1.331) | 1.046(0.842-1.299) | 1.045(0.914-1.193) |
| DROSHA rs2291109(A>T) | | | | | |
| Overall | 0.922(0.765-1.110) | 1.014(0.771-1.333) | 0.932(0.770-1.128) | 1.046(0.798-1.371) | 0.958(0.815-1.126) |
| Yue Jiang | 0.949(0.702-1.284) | 1.233(0.875-1.737) | 0.978(0.740-1.294) | 1.258(0.897-1.765) | 1.016(0.836-1.234) |
| Yue Jiang | 0.839(0.723-0.975) | 0.890(0.635-1.248) | 0.845(0.732-0.975) | 0.949(0.680-1.325) | 0.883(0.783-0.995) |
| Lin Yuan | 0.977(0.770-1.240) | 0.958(0.690-1.330) | 0.975(0.738-1.289) | 0.967(0.699-1.338) | 0.978(0.752-1.272) |
| DGCR8 rs3757(G>A) | | | | | |
| Overall | 1.022(0.750-1.391) | 0.894(0.460-1.737) | 1.005(0.748-1.350) | 0.885(0.460-1.704) | 0.986(0.772-1.260) |
| Jong-Sik Kim | 0.994(0.700-1.411) | 0.781(0.344-1.771) | 0.968(0.690-1.358) | 0.783(0.349-1.756) | 0.949(0.717-1.256) |
| Yohei Horikawa | 1.125(0.584-2.168) | 1.167(0.370-3.676) | 1.133(0.617-2.081) | 1.126(0.364-3.490) | 1.116(0.675-1.845) |
| DGCR8 rs417309(G>A) | | | | | |
| Overall | 1.282(1.057-1.555) | 3.169(1.634-6.146) | 1.365(1.131-1.647) | 3.026(1.574-5.817) | 1.423(1.196-1.693) |
| Antoni Bruzgielewicz | 1.291(1.054-1.582) | 3.040(1.366-6.765) | 1.352(1.108-1.649) | 2.911(1.322-6.408) | 1.391(1.155-1.676) |
| Yue Jiang | 1.214(0.957-1.540) | 3.169(1.634-6.146) | 1.338(1.065-1.681) | 3.026(1.574-5.817) | 1.432(1.166-1.760) |
| Yue Jiang | 1.206(0.963-1.510) | 3.157(1.579-6.310) | 1.305(1.050-1.622) | 3.009(1.520-5.954) | 1.381(1.133-1.684) |
| Jong-Sik Kim | 1.302(1.069-1.585) | 3.169(1.634-6.146) | 1.388(1.146-1.681) | 3.026(1.574-5.817) | 1.446(1.212-1.726) |
| Ewa Osuch-Wojcikiewicz | 1.293(1.052-1.590) | 3.525(1.368-9.081) | 1.353(1.104-1.657) | 3.402(1.332-8.691) | 1.394(1.150-1.689) |
| Yohei Horikawa | 1.361(1.105-1.676) | 3.082(1.538-6.178) | 1.442(1.177-1.767) | 2.925(1.474-5.806) | 1.490(1.235-1.796) |
| DGCR8 rs1640299(T>G) | | | | | |
| Overall | 0.895(0.645-1.243) | 0.998(0.751-1.325) | 0.898(0.659-1.223) | 0.965(0.745-1.249) | 0.959(0.852-1.078) |
| Antoni Bruzgielewicz | 1.008(0.762-1.333) | 1.054(0.786-1.415) | 1.004(0.802-1.259) | 0.990(0.758-1.292) | 0.991(0.877-1.120) |

| | | | | | |
|---|---|---|---|---|---|
| Yue Jiang | 0.837(0.464-1.508) | 1.050(0.696-1.584) | 0.840(0.482-1.465) | 0.956(0.674-1.356) | 0.953(0.790-1.150) |
| Jong-Sik Kim | 0.926(0.617-1.390) | 0.984(0.735-1.318) | 0.911(0.615-1.349) | 0.946(0.726-1.233) | 0.962(0.852-1.087) |
| Yohei Horikawa | 0.784(0.556-1.106) | 0.906(0.637-1.289) | 0.790(0.559-1.116) | 0.964(0.684-1.360) | 0.918(0.801-1.052) |

Note: OR, odds ratio; CI, confidence interval.
